# Mesenchymal Stem Cell Seeding of Porcine Small Intestinal Submucosal Extracellular Matrix for Cardiovascular Applications

**DOI:** 10.1371/journal.pone.0153412

**Published:** 2016-04-12

**Authors:** Chia Wei Chang, Tye Petrie, Alycia Clark, Xin Lin, Claus S. Sondergaard, Leigh G. Griffiths

**Affiliations:** 1 Department of Medicine and Epidemiology, School of Veterinary Medicine, University of California, Davis, Davis, California, United States of America; 2 Department of Surgery, School of Medicine, University of California, Davis, Sacramento, California, United States of America; Michigan Technological University, UNITED STATES

## Abstract

In this study, we investigate the translational potential of a novel combined construct using an FDA-approved decellularized porcine small intestinal submucosa extracellular matrix (SIS-ECM) seeded with human or porcine mesenchymal stem cells (MSCs) for cardiovascular indications. With the emerging success of individual component in various clinical applications, the combination of SIS-ECM with MSCs could provide additional therapeutic potential compared to individual components alone for cardiovascular repair. We tested the *in vitro* effects of MSC-seeding on SIS-ECM on resultant construct structure/function properties and MSC phenotypes. Additionally, we evaluated the ability of porcine MSCs to modulate recipient graft-specific response towards SIS-ECM in a porcine cardiac patch *in vivo* model. Specifically, we determined: 1) *in vitro* loading-capacity of human MSCs on SIS-ECM, 2) effect of cell seeding on SIS-ECM structure, compositions and mechanical properties, 3) effect of SIS-ECM seeding on human MSC phenotypes and differentiation potential, and 4) optimal orientation and dose of porcine MSCs seeded SIS-ECM for an *in vivo* cardiac application. In this study, histological structure, biochemical compositions and mechanical properties of the FDA-approved SIS-ECM biomaterial were retained following MSCs repopulation *in vitro*. Similarly, the cellular phenotypes and differentiation potential of MSCs were preserved following seeding on SIS-ECM. In a porcine *in vivo* patch study, the presence of porcine MSCs on SIS-ECM significantly reduced adaptive T cell response regardless of cell dose and orientation compared to SIS-ECM alone. These findings substantiate the clinical translational potential of combined SIS-ECM seeded with MSCs as a promising therapeutic candidate for cardiac applications.

## Introduction

Several animal and clinical studies have demonstrated the ability of decellularized porcine SIS-ECM to mediate tissue repair in a range of regenerative applications, including wound healing [1–4], bladder regeneration [5–7], tendon graft [8] gastrointestinal grafts [9–11] and cardiovascular repairs [12–18]. The clinical success of this collagen-rich biomaterial has been suggested to correlate with its micro three-dimensional ECM structural environment [19], bioactive molecules within the material [20] and its biodegradability which fosters integration with host tissue [14]. Additionally, matrix-derived cell signaling molecules (cytokines and growth factors) have been demonstrated to play an important role in modulating fibrosis [2], inflammation [21, 22] and promoting angiogenesis [19, 23–25] which can be critical to mediate tissue regenerative responses.

Clinically, SIS-ECM patches have been utilized for surgical correction of congenital cardiovascular defects, including pericardial, aortic and pulmonary artery reconstruction, vascular and septal defect restoration, as well as valvular repair [12–18]. These studies demonstrate SIS-ECM compatibility with host cardiovascular tissues to provide structural support and potential for enhancement of regenerative responses to repair cardiovascular defects. Simultaneously, cellular therapies for cardiac regenerative medicine have been investigated for many years with promising results. In particular, the multi-potent bone marrow-derived MSCs have been utilized for treatment of myocardial infarct (MI) in animal studies where they exhibited the ability to foster cardiovascular regeneration through paracrine signaling pathways [26, 27]. Specifically, the mechanism by which MSCs modulate vascular and cardiac tissue repair have been associated with release of a diverse range of pro-angiogenic, pro-migratory, pro-survival and immunomodulatory cytokines, capable of modulating local effector cell function [28, 29].

In addition, several clinical trials have examined the therapeutic potential of MSCs and various injection delivery routes for ischemic cardiac injury in patients [30–33]. Such studies reported initially promising outcomes demonstrating feasibility and safety of cell delivery methods, with positive local regenerative responses. However, these catheter-based delivery methods failed to show long-term retention of delivered cells, thereby reducing the potential of MSCs to mediate cardiovascular regeneration. Harnessing the potential synergistic effects of a bioactive SIS-ECM matrix with the immunomodulatory and pro-regenerative properties of MSCs has the potential to further improve the therapeutic outcome for patients by providing an alternative delivery method for MSCs. While the use of SIS-ECM to deliver MSCs to the local injured tissue site has been explored in several *in vivo* animal studies, such as urinary bladder augmentation [6, 7, 34, 35], tracheal reconstruction [36], skin wound healing [2], as well as cervical and abdominal grafts [9, 10], very little is known regarding the effect of SIS-ECM combined with MSC delivery for cardiovascular indications. Recent studies examining the effects of SIS-ECM in promoting MSCs proliferation, differentiation and angiogenic cytokine secretions (vascular endothelial growth factor and interleukin-8) further highlight the importance of appropriate biomaterial selection for cardiovascular applications [37, 38]. The motivation for the current study is therefore to examine the translational potential of MSC-seeded SIS-ECM as a vehicle for delivery of both effector cells and bioactive matrix for cardiovascular regenerative applications.

The goals of this study are to determine: 1) maximal loading capacity of SIS-ECM with MSCs, 2) biochemical composition, mechanical and structural properties of the combined MSC-seeded SIS-ECM biomaterial, 3) phenotypes of MSCs pre- and post-seeding on SIS-ECM, and 4) target MSC dose and orientation to be used in an *in vivo* epicardial cardiac application. Prior to using hMSC-seeded SIS-ECM constructs for cardiovascular tissue repairs, it is critical to determine if hMSC-seeding of SIS-ECM results in any detrimental effects on either component of the resultant construct. We hypothesize that hMSC-seeding will not alter the SIS-ECM biomaterial structure, mechanical function and biochemical compositions. Similarly, we hypothesize that the cellular phenotype and differentiation potential of MSCs are maintained post-seeding onto SIS-ECM. Lastly, we hypothesize that an optimal MSC dose and orientation of seeded patch *in vivo* will enhance the immunomodulatory properties of MSCs on SIS-ECM patch for cardiovascular applications.

## Materials and Methods

### Animals

All animal procedures were conducted in accordance with the guidelines established by the Guide for the Care and Use of Laboratory Animals [39]. The protocol was approved by the Institutional Animal Care and Use Committee (IACUC) at University of California, Davis (approval number: 17674).

### Reagents and materials

High glucose Dublecco’s modified eagle medium (DMEM), L-glutamine 200 mM (100x), penicillin/streptomycin solution and phosphate-buffered saline (PBS) were purchased from Hyclone Laboratories (South Logan, UT). Fetal bovine serum (FBS) premium select was purchased from Atlanta Biologicals (Lawrenceville, GA). 96-well tissue culture plates were from Corning Inc. (Corning, NY).

### Human MSCs and porcine MSCs isolation

Human bone marrow (Lonza, Allendale NJ) or porcine bone marrow samples (Yucatan mini-pigs, S & S Farms, Ramona, CA) were diluted 1:3 and plated in high glucose DMEM supplemented with 1% L-glutamine, 20% FBS and 1% penicillin/streptomycin in T75 flasks and cultured overnight at 37°C with 5% CO_2_. Next day, non-adherent cells were removed, and flask was gently rinsed with sterile PBS three times, followed by addition of fresh culture medium. Each flask was allowed to reach 80% confluence before passaging.

### Labeling of human MSCs and imaging

The human MSCs (hMSCs) were transduced with a green fluorescent protein (GFP) expressing lentiviral vector and seeded onto 6 mm diameter circular SIS-ECM discs. The images were recorded on a Motic AE30-31 inverted fluorescence microscope at the magnification indicated using a Moticam Pro252A imaging system (both Motic Instruments, Canada). Matrices were inverted before imaging to allow visualization of the cells.

### Characterization of human MSCs and porcine MSCs

Cells were analyzed by flow cytometry (Coulter FC500, Beckman Coulter, Brea CA) using the following antibodies: 1) human samples—CD34, CD19, HLA-DR, CD90 (all BD Biosciences, San Jose CA), CD44 (both Biolegend, San Diego CA), CD14, CD45 (AbB Serotec, Raleigh NC), CD105 (Abcam, Cambridge, MA); 2) porcine samples–CD44 (Biolegend), CD90 (antibodies-online.com Atlanta, GA), CD105 (Abcam), CD79a, CD45, SLA-DR (all AbD Serotec). Matched isotype controls were analyzed in parallel.

### Tri-lineage induction of human MSCs and porcine MSCs

Cells for adipogenic and osteogenic induction were seeded on normal tissue culture wells while cells for chondrogenic cultures were seeded as micromass cultures in round bottom tubes. Control cells received normal growth medium while induction cultures (osteogenic, adipogenic and chondrogenic) received growth media with supplements according to the manufactures recommendations (Lonza). Medium was changed every 2–3 days for both induced and control cultures. Adipocytes and osteocytes were visualized by Oil Red O and Alizarin Red staining, respectively, while chondrogenic cultures were stained by Alcian Blue staining following cryosectioning. All images were recorded on a Motic AE30-31 inverted fluorescence microscope at the magnification indicated using a Moticam Pro252A imaging system (both Motic Instruments, Canada).

### MTT assay to determine MSC-loading capacity on SIS-ECM

To determine viability, hMSCs were seeded at 10,000, 50,000, 100,000 and 150,000 cells per well or porcine MSCs (pMSCs) seeded at 20,000, 100,000, 200,000 and 300,000 cells per well. The cells were plated either on a 6 mm diameter disc of porcine SIS-ECM or on the plastic surface of the 96-well plate and cultured overnight. Cell culture medium was removed and replaced with 100 μl fresh medium and 20 μl MTT in PBS (5 mg/ml). Cells were incubated for 3.5 h at 37°C with 5% CO_2_. Medium was carefully removed and replaced with 150 μl MTT solvent containing 0.1% Nonidet P-40 and 4 mM HCl. The plate was covered with foil and agitated on an orbital shaker for 15 min. Solubilized MTT was transferred to a fresh 96-well plate and absorbance read at 570 nm with 650 nm as a reference wavelength. For each data point, the experiment was done in triplicates, and each MTT assay repeated three times.

### SIS-ECM seeding with human MSCs and porcine MSCs

#### *In vitro* assessment

All experiments were performed using hMSCs or pMSCs between passage 3 and 7. SIS-ECM samples were prepared as 6 mm discs of various seeding densities for MTT assay (see above) or as 3 x 10 mm strips (histology, biochemistry and tensile test) seeded with hMSCs at 350,000 cells/cm^2^. Seeded SIS-ECM samples and non-seeded controls were cultured up to 96 h at 37°C with 5% CO_2_ before each experiment. An additional rehydrated SIS-ECM sample (soaked in medium for 30 min) was also included in all experiments.

#### *In vivo* assessment

*In vivo* porcine study (n = 6) was performed using 6 mm diameter discs of SIS-ECM seeded with pMSCs (passage 3–7) at low (65,000 cells/cm^2^) or high (650,000 cells/cm^2^) density. The seeded SIS-ECM discs were cultured for 24 h before being introduced in a porcine *in vivo* epicardial study.

### Post-seeding analysis of porcine MSCs

To assess the tri-lineage differentiation potential, pMSCs were cultured on SIS-ECM or on plastic for 3 days, and recovered by gentle trypsin digestion (5 min), followed by sub-culture until 80% confluence. The tri-lineage differentiation potential and cell surface marker expression were re-assessed as described above.

### Histology

All samples were fixed in 10% formalin solution for 24 h prior to processing for hematoxylin and eosin (H&E) and Verhoeff Van Geison (VVG) stains. Fixed samples were embedded in paraffin and sectioned for staining, and representative images were captured with an upright microscope (Nikon Eclipse E600) with a 40x objective lens.

### Scanning electron microscopy (SEM)

Samples were fixed in Karnovsky’s solution (2.5% paraformaldehyde, 2% glutaraldehyde in 0.08 M sodium phosphate buffer, pH 7.2) overnight at 4°C prior to SEM processing. Samples were rinsed with 0.1 M sodium phosphate buffer three times with 5 min incubation. All samples were dehydrated in ascending concentrations of ethanol (30%, 50%, 70%, 95% (3x) and 100% (3x)) for 10 min each. Following dehydration, the samples were subjected to critical point dry (Tousimis 931 GL Super Critical Autosamdri, Tousimis Research Corp., Rockville, MD). Post-drying, samples were mounted onto aluminum stubs and coated with gold (Pelco Auto Sputter Coater SC-7, Ted Pella Inc., Redding, CA). SEM images were captured using 10 kV on scanning microscope (Philips XL30 TMP, FEI Company, Hillsborow, OR).

### Uniaxial tensile testing

A dogbone shape was created using a 2 mm biopsy punch (Miltex) to subtract a semicircle from the midpoint of each of the long sides in the 3 x 10 mm SIS-ECM samples. Initial gauge length was therefore defined as 2 mm (S1 Fig). Thickness of the dogbone region was measured using digital calipers. Width of the dogbone region was measured on digital images using ImageJ (NIH), and samples were loaded onto Instron unit (model 5965) equipped with Bluehill 3 software. Each sample was subjected to a constant strain rate (0.1 mm/s), and resultant stress-strain curves were generated. Failure mode for all samples was recorded. Young’s modulus and ultimate tensile stress (UTS) were calculated for each sample.

### Quantitative biochemistry

Following seeding, sample wet weight was recorded and followed by freezing at -20°C overnight before lyophilization for 72 h. Post-lyophilization, dry weight (DW) of each sample was recorded. Pyridinoline (PYR) crosslinks of SIS-ECM were quantified using high performance liquid chromatography as previously described [40]. Briefly, samples of SIS-ECM wet weight were recorded, followed by digestion in 6 N HCl and dried using a vacuum to concentrate the samples. 50 μl of solution with 10 nM pyridoxine and 2.4 μmol homoarginine was added to resuspend the sample and diluted 5-fold with a solution containing 0.5% heptaluorobutyric acid in 10% acetonitrile. All samples were injected into C18 column (Shimadzu, Columbia, MD) and eluted as a solvent profile described previously [41]. The PYR crosslinks of each sample were quantified accordingly with a reference curve using PYR standards (Quidel, San Diego, CA). Collagen content per DW was calculated following papain digest using a modified colorimetric hydroxyproline assay as previously described [42]. Elastin per DW was quantified following 0.25 M oxalic acid digest using a Fastin elastin assay (Biocolor Ltd, UK). Sulfated glycosaminoglycan (sGAG) per DW was measured after papain digestion using a Blyscan GAG assay kit (Biocolor Ltd, UK).

### Porcine *in vivo* epicardial patch model

Porcine MSC-dosing and orientation studies were conducted using 6-month old male, Yucatan mini-pigs (n = 6). Pigs were anesthetized, and the heart approached using a standard midline mini-thoracotomy. SIS-ECM discs were prepared with high dose (650,000 cells/cm^2^) or low dose (65,000 cells/cm^2^) of pMSCs and implanted with cells oriented either toward or away from the epicardium (S1 Table). In each pig, five individual 6 mm discs of SIS-ECM were sutured to the epicardial surface of the left ventricular free wall. Discs were oriented in a known sequence in each pig, with the left anterior descending coronary artery, first and second diagonal arteries used as anatomic landmarks to aid in later identification of each disc. After 2 weeks, mini-pigs were euthanized, hearts explanted and each of the five discs isolated en-bloc with its underlying myocardium. All samples were fixed in 10% formalin for 24 h prior to paraffin embedding and processing for H&E and immunohistochemistry staining.

### Immunohistochemistry (IHC) staining

Groups of sectioned unstained slides from each disc were de-parafiinized with xylene (Fisher Scientific) and rehydrated with an ethanol gradient prior to each IHC staining protocol. Briefly, endogenous peroxidases were blocked with 3% hydrogen peroxide in methanol (Fisher Scientific) at room temperature (RT) for 15 min, followed by antigen retrieval with steamed target retrieval solution (pH = 9) (Dako) for 30 min. Slides were washed three times (2 min) with phosphate buffer saline supplemented with 0.01% tween solution (PBST) and blocked with 4% non-fat milk (Lab Scientific) in PBST for 30 min at RT. Primary antibody was applied at desired dilution and incubated overnight at 4°C. Next day, slides were washed with PBST (5 x 2 min), and secondary peroxidase-labelled polymer (Dako) was applied for 30 min at RT. Presence of bound primary antibody was detected using EnVision DAB+chromogen (Dako) for 10 min at RT. Lastly, slides were counterstained with Mayer’s hematoxylin (Fisher Scientific) for 10 min, rinsed under running tap water for 2 min, dehydrated with an ethanol gradient, cleaned with xylene and mounted with Permount (Fisher Scientific) before being coverslipped. Specific antibodies used were: CD3 (1:200) (Abcam, ab16669) for T cell marker, CD79a (1:1000) (Abcam, ab3121) for B cell marker, and CD31 (1:50) (Abcam, ab28364) for endothelial cell marker. Following each staining protocol, slides were visualized and images were captured with a light microscope and 4x objective lens (Nikon). Next, cells stained for CD3 and CD79a were quantified using NIS Elements (Nikon, version 4.30). A region of interest (ROI) around the scaffold was determined, and a color saturation threshold was established as a parameter for the software to identify positively stained cells (brown coloration). The percentage of area above the threshold over the total area of ROI was measured and calculated. The mean and standard deviation (SD) for each condition were quantified.

### Statistical analysis

Data were presented as mean ± SD. One-way analysis of variance (ANOVA) was performed across all groups with Bonferroni or Tukey’s *post hoc* test using Prism GraphPad or JMP software to determine statistical significance (p < 0.05). Groups not connected by the same letter were statistically significant.

## Results

### Maximum cell loading density of SIS-ECM

**Fig 1:**
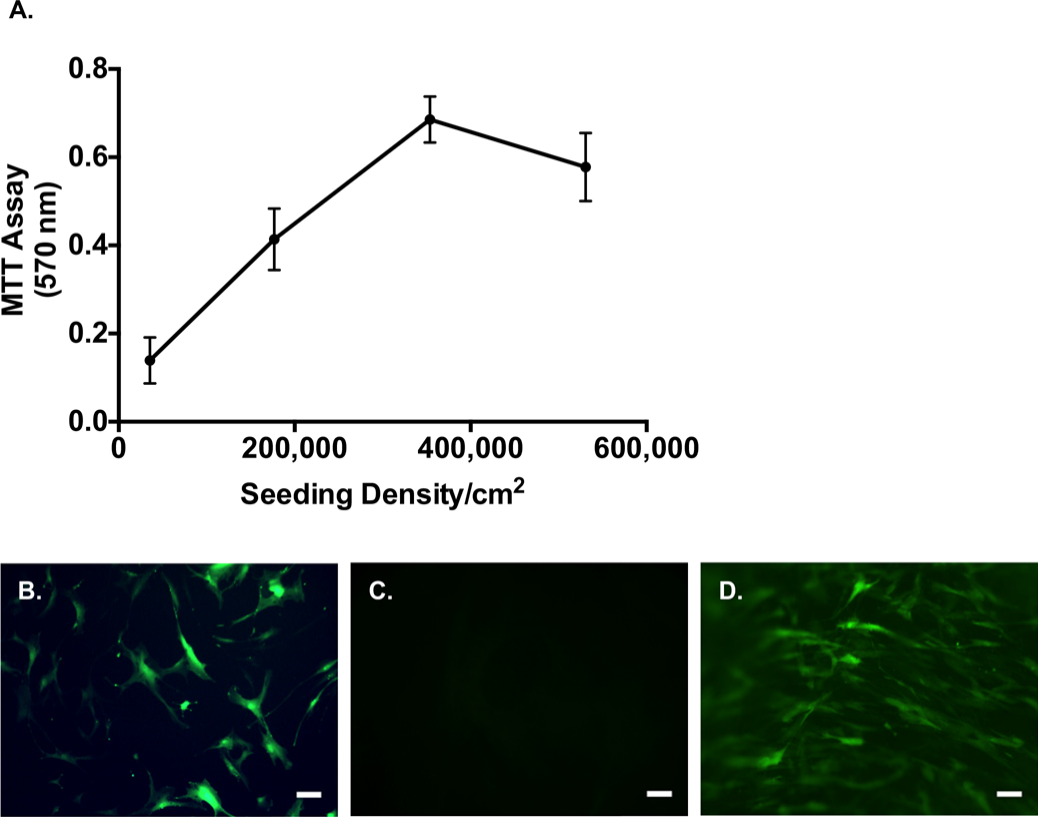
Maximum Cell Loading Density of SIS-ECM. (A) Increasing loading densities of hMSCs on SIS-ECM were assessed, and viability 24 h post-seeding was measured. Data points were represented as mean ± SD at each seeding density. Maximum hMSC-loading density of SIS-ECM was determined to be 350,000 cells/cm^2^. (B) Fluorescence image of eGFP-labled hMSCs cultured *in vitro* on tissue culture plastic. (C) SIS-ECM without eGFP-labeled hMSC-seeding showed minimal background signal. (D) Fluorescence image of eGFP-labeled hMSCs seeded and cultured on SIS-ECM. Scale bar denotes 50 μm.

We first confirmed that cells isolated from human bone marrow were MSCs based on their cell surface marker expressions (positive for CD44, CD90 and CD105 and negative for hematopoietic and endothelial markers), and tri-lineage differentiations into adipocytes, osteocytes and chondrocytes (data not shown). Next, we determined the maximum cell loading capacity of SIS-ECM via MTT assay to determine the maximum number of viable cells deliverable using SIS-ECM as a vehicle. A loading dose-dependent increase in adhesion of viable cells was found at cell doses up to and including 350,000 cells per cm^2^ (Fig 1A). Above this seeding density, no further increase in viable cell adherence was achieved on SIS-ECM. We therefore chose 350,000 cells per cm^2^ as the seeding density in the subsequent experiments for hMSCs, as this represented the maximal number of adherent hMSCs that the SIS-ECM could support. To confirm that hMSCs can be seeded onto the SIS-ECM biomaterial, the cells were transduced with eGFP using a lentiviral vector and cultured on normal tissue plastic or SIS-ECM. The eGFP-labeled hMSCs could be visualized using fluorescent microscopy, as a monolayer on normal tissue culture plastic (Fig 1B). Background fluorescence from control SIS-ECM was minimal (Fig 1C), and seeded eGFP-labeled hMSCs were observed to adhere on the three-dimensional SIS-ECM (Fig 1D).

### Histology

**Fig 2:**
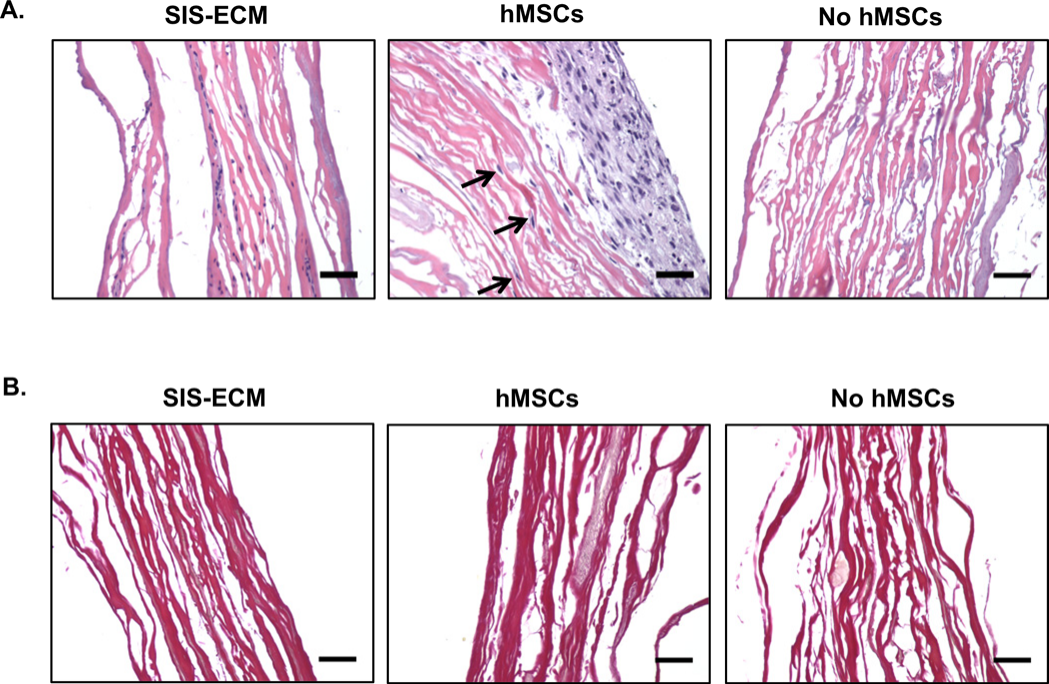
Histology of SIS-ECM. (A) H&E staining of SIS-ECM in medium for 30 min (left), non-seeded SIS-ECM (right) and SIS-ECM seeded with hMSCs (middle). Seeded hMSCs were observed to grow in layers on the surface of SIS-ECM with occasional migration within the matrix (denoted by arrows). (B) SIS-ECM was composed of mostly collagen and minimal elastin based on VVG staining. Scale bar denotes 10 μm.

Histological H&E staining showed that there were noticeable nuclei present in the collagenous SIS-ECM material (Fig 2A). However, SIS-ECM without hMSC-seeding had loss of remnant nuclei after 96 h of culture (Fig 2A). In the seeded group, hMSCs were observed to adhere mostly on the surface of SIS-ECM, with minimal migration into material between collagen fibers. Qualitatively, porcine SIS-ECM contained mostly collagen fibers with minimal elastin, which when present was predominantly associated with blood vessel based on VVG staining (Fig 2B). Seeding with or without hMSCs did not alter the gross histologic morphology of SIS-ECM biomaterial.

### SEM analysis

**Fig 3:**
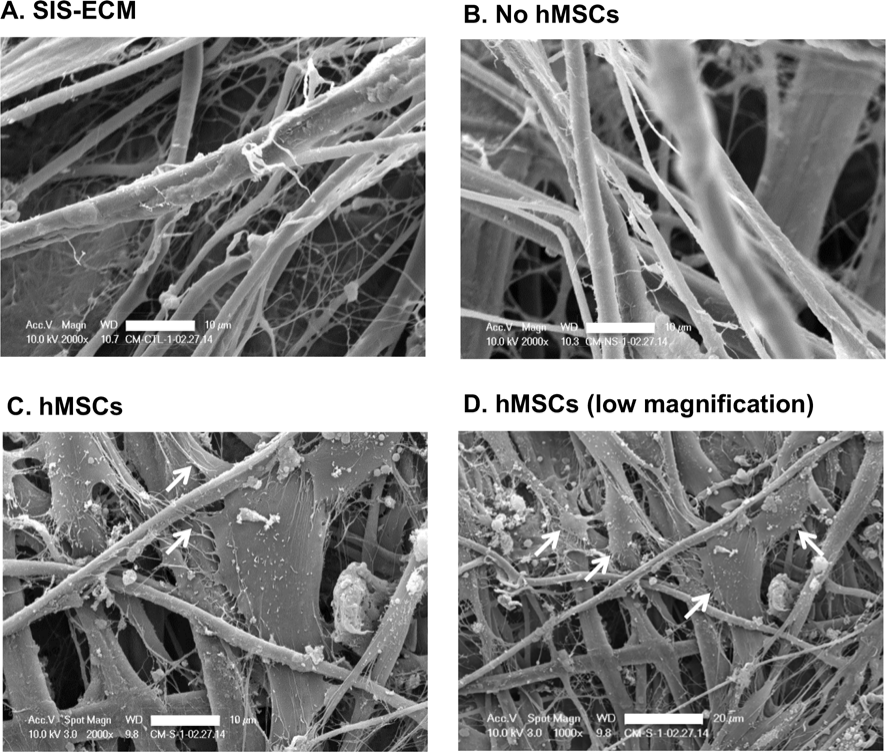
Representative SEM Images of SIS-ECM. (A) Porcine SIS-ECM contained well-preserved collagen fibers in sample rehydrated in medium after 30 min and (B) following 96 h culture. (C) Post-seeding, hMSCs were observed to adhere and grow between collagen fibers with visible cell protrusions (denoted by white arrows) attaching to SIS-ECM. (D) Low magnification of seeded hMSCs revealed colony clusters of hMSCs on SIS-ECM. Scale bar denotes 10 μm.

We used SEM to examine the structure of SIS-ECM and the effect of hMSC-seeding. The collagen fibrous network was maintained between SIS-ECM, non-seeded and seeded groups (Fig 3A–3C). Post-seeding, hMSCs were observed to adhere and grow between collagen fibers with visible cell protrusions attaching to the collagen fibrous network of SIS-ECM (Fig 3C and 3D). At lower magnification, seeded group showed that multiple hMSCs were able to colonize the surface of SIS-ECM (Fig 3D). The SEM images showed that SIS-ECM contained layers of intact collagen fibers, and it was capable of supporting hMSC repopulation and growth in culture.

### Uniaxial tensile testing

**Fig 4:**
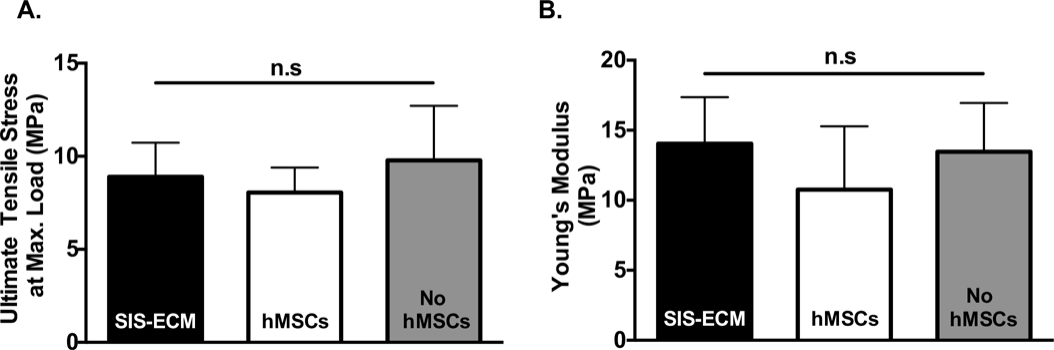
Uniaxial Tensile Testing of SIS-ECM. (A) Data were plotted as mean ± SD for UTS (n = 8) and (B) Young’s modulus (n = 6). Mechanical properties of SIS-ECM were maintained before and after hMSC-seeding.

Uniaxial tensile testing was performed to examine the mechanical integrity of SIS-ECM and effect of hMSC-seeding. All samples were observed to fail at the dogbone region. No statistical significance was detected for UTS and Young’s modulus between SIS-ECM group (8.90 ± 1.83 MPa and 14.04 ± 3.32 MPa), hMSC-seeded group (8.05 ± 1.34 MPa and 10.76 ± 4.52 MPa) or non-seeded group (9.78 ± 2.94 MPa and 13.47 ± 3.48 MPa) (p = 0.2953 for UTS and p = 0.3107 for Young’s modulus) (Fig 4A and 4B).

### Biochemical analysis

**Fig 5:**
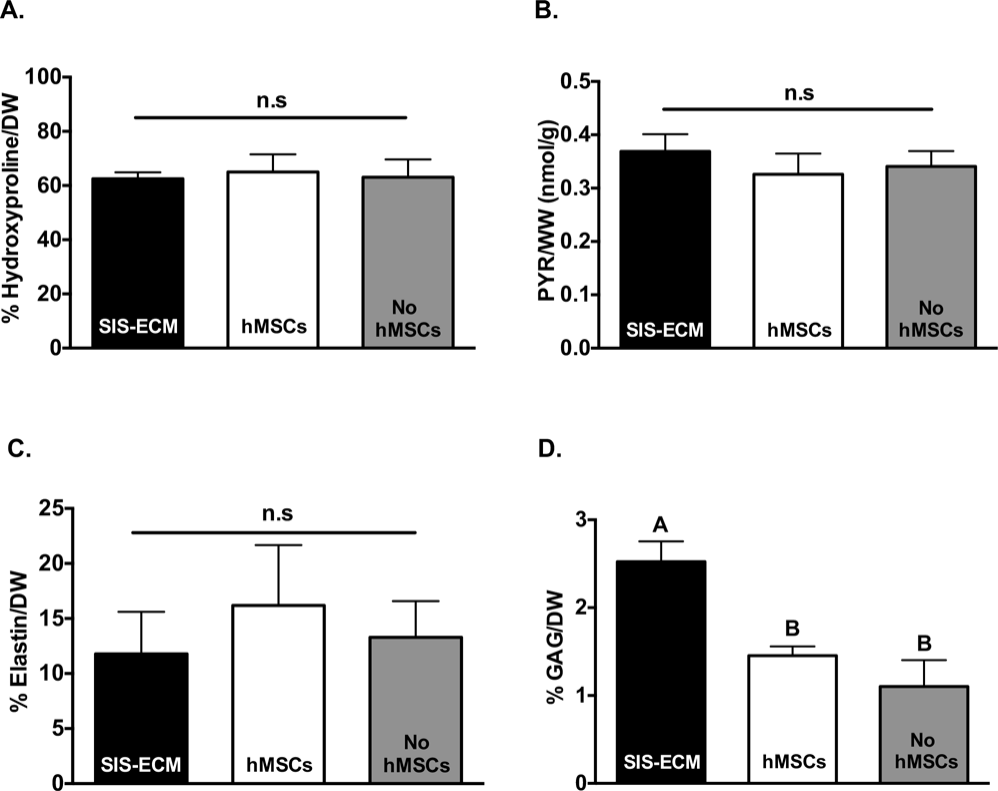
Quantitative Biochemistry of SIS-ECM. (A) Percent collagen content (n = 6), (B) collagen pyridinoline crosslinking (n = 6) and (C) elastin content (n = 12) were maintained following culture with or without hMSC-seeding. (D) GAG content (n = 6) was reduced in culture and post-seeding of hMSCs. Data were plotted as mean ± SD.

Biochemical content of collagen, elastin and sGAG were analyzed to assess the effect of hMSC-seeding on SIS-ECM compositions. Biochemical analysis of hydroxyproline indicated no significant difference in collagen content between SIS-ECM (62.51 ± 2.40% per DW), hMSC-seeded (64.99 ± 6.51% per DW) and non-seeded groups (63.03 ± 6.61% per DW) (p = 0.7192) (Fig 5A). Additionally, no significant difference was detected in collagen pyridinoline crosslinking between SIS-ECM (0.37 ± 0.03 nmol/g), hMSC-seeded (0.33 ± 0.04 nmol/g) and non-seeded groups (0.34 ± 0.03 nmol/g) (p = 0.1144) (Fig 5B). Elastin content of SIS-ECM was not significantly different between hMSC-seeded (16.19 ± 5.49% per DW), non-seeded group (13.28 ± 3.30% per DW) and SIS-ECM group (11.78 ± 3.83% per DW) (p = 0.0513) (Fig 5C). Quantified sGAG per DW in SIS-ECM group (2.52 ± 0.23%) was significantly higher than hMSC-seeded (1.45 ± 0.11%) and non-seeded groups (1.10 ± 0.30%) (p < 0.0001) (Fig 5D). However, sGAG content was not statistically different between hMSC-seeded and non-seeded groups.

### Seeding and characterization of porcine MSCs

**Fig 6:**
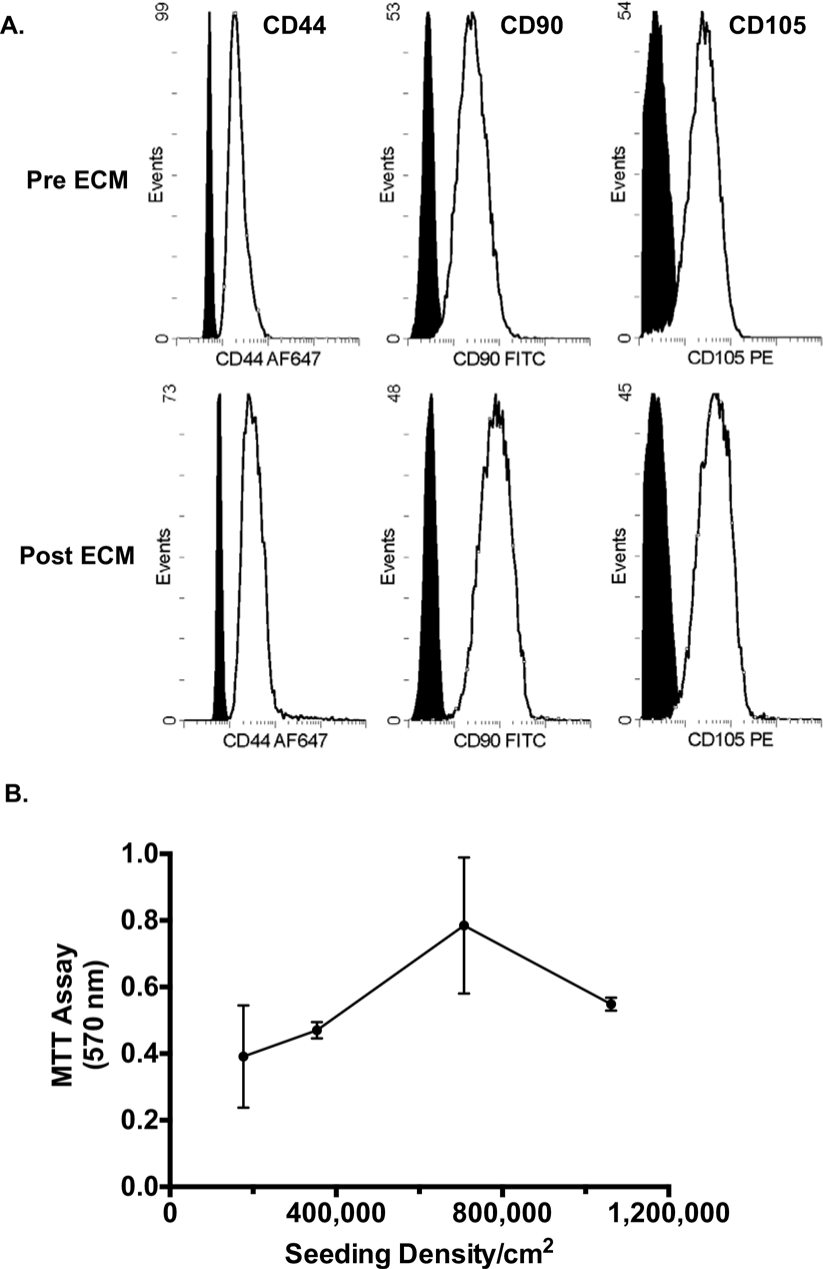
Characterization and Maximum Loading Density of Porcine MSCs on SIS-ECM. (A) Putative MSC surface markers for isolated cells from porcine bone marrow (CD44, CD90 and CD105) were characterized on flow cytometry before seeding on SIS-ECM (Pre ECM) and after culture (Post ECM). (B) Maximum loading capacity of pMSCs on SIS-ECM was determined using MTT assay to assess viability. Data points were represented as mean ± SD at each seeding density. Maximum pMSC loading density of porcine SIS-ECM was determined to be 700,000 cells/cm^2^.

To facilitate the assessment of the *in vivo* application of seeded SIS-ECM in a porcine epicardial model, we developed a porcine analogue of the hMSC-seeded SIS-ECM construct. Isolated porcine cells were first characterized based on their cell surface marker expressions (CD44, CD90 and CD105) (Fig 6A) and tri-lineage differentiation potential to establish their MSC identity (S2 Fig). Subsequently, we also confirmed that post-seeding, SIS-ECM did not negatively affect the phenotypes (Fig 6A) and differentiation potential of porcine MSCs (S2 Fig). In addition, maximum porcine MSC-loading capacity of SIS-ECM was determined to be 700,000 cells per cm^2^ without negatively affecting cell viability (Fig 6B). This loading density information was used to seed two target doses (high vs. low) of pMSCs for the following *in vivo* patch material preparation.

### Porcine *in vivo* patch model

**Fig 7:**
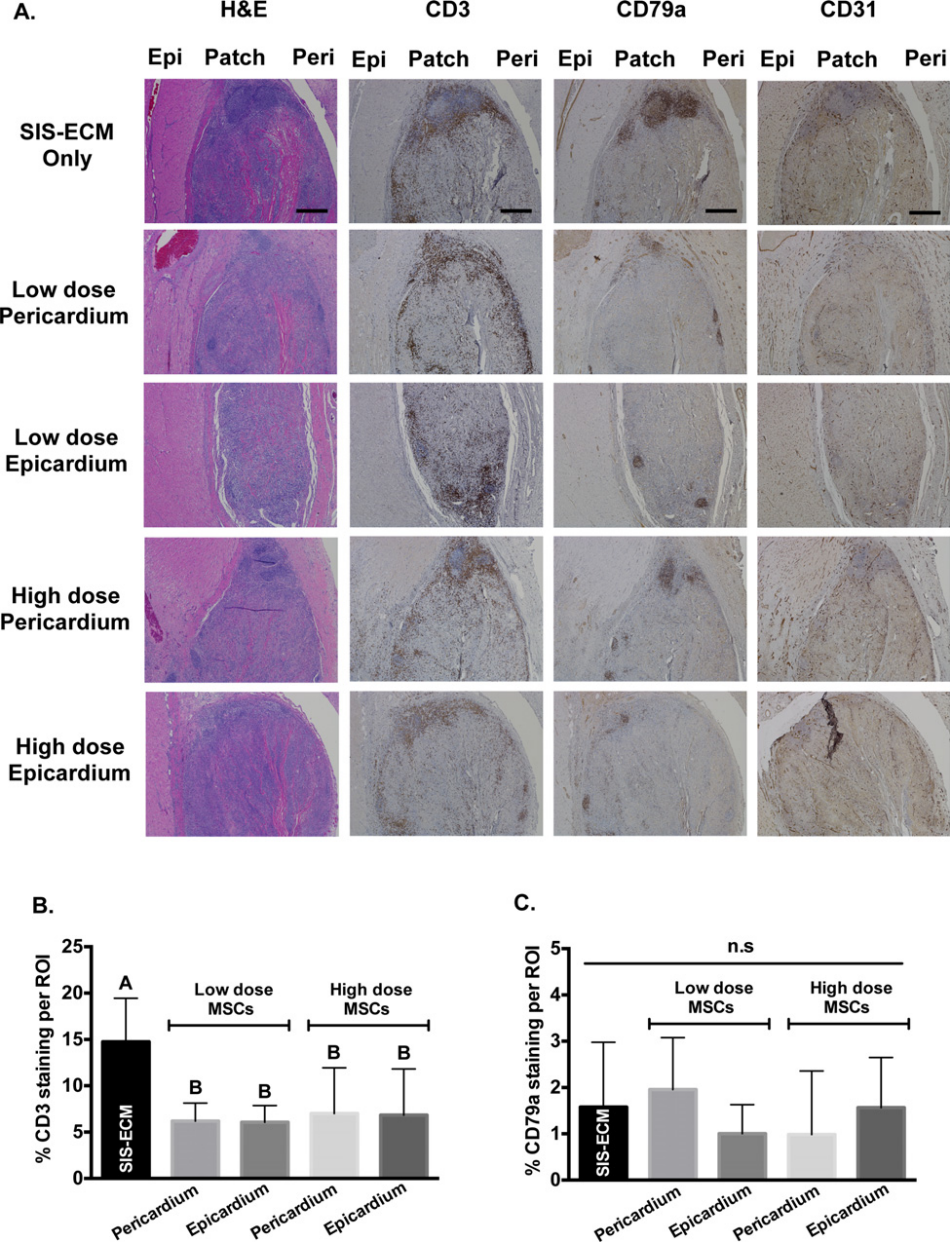
Histology and Immune Response of Porcine *In Vivo* Patch Study. (A) Representative H&E staining, CD3 staining for T cell marker, CD79a staining for B cell marker and CD31 staining for endothelial cells of SIS-ECM treatment groups. (B) T cell (CD3 staining) response was significantly reduced with MSC-seeding compared to SIS-ECM patch alone. (C) B cell (CD79a staining) numbers were comparable across all groups. Scale bar denoted 500 μm.

To assess the translational potential of an MSC-seeded SIS-ECM patch, we evaluated two target doses representing a low and a high seeding density, as well as orientation of seeded cells relative to the epicardium. We used 6 mm discs seeded with pMSCs during a two-week *in vivo* porcine epicardial patch model. We tested if high (650,000 cells/cm^2^) or low (65,000 cells/cm^2^) dose of pMSCs and their orientation towards (epicardial) or away (pericardial) from the epicardium altered *in vivo* response towards the FDA-approved SIS-ECM biomaterial. Overall, inflammatory response was similar across all groups. However, low dose pMSCs resulted in decreased scarred tissue formation compared to high dose or patch alone groups (Fig 7A). Qualitatively, endothelial cell staining with CD31 had similar amount of blood vessel formation present within and surrounding the SIS-ECM regardless of pMSC presence, dosage or orientation (Fig 7A). Adaptive immune response was reduced in cell-seeded SIS-ECM compared to SIS-ECM patch only, although this finding was predominantly due to reduction in T cell response (CD3 staining) with B cell infiltration (CD79a staining) largely unaffected. Quantification of percentage area of T cell numbers (CD3 staining) around the scaffold demonstrated that MSC-seeding groups (low dose and high dose) showed a reduction in T cell staining regardless of orientation compared to SIS-ECM patch alone (p = 0.0023) (Fig 7B). In contrast, B cell (CD79a staining) quantification yielded no statistical significance across all treatment groups (p = 0.3981) (Fig 7C).

## Discussion

In this study, we examined the structure and function properties of porcine SIS-ECM before and after hMSC-seeding *in vitro* to investigate the potential clinical translation of such a seeded biomaterial. We determined that the maximum loading densities of hMSCs and pMSCs that can be supported on SIS-ECM without affecting cell viability were 350,000 cells/cm^2^ and 700,000 cells/cm^2^ respectively. The biochemical components of SIS-ECM were largely unaffected by hMSC-seeding, with collagen, elastin and collagen crosslinking preserved during *in vitro* studies. However, sGAG content was decreased during culture, although this component represented a small fraction of the material’s composition (< 3% per DW). Finally, mechanical properties of SIS-ECM were maintained following hMSC-seeding. Seeding of pMSCs on SIS-ECM did not alter their cell surface marker expression or tri-lineage differentiation potential. Furthermore, a two-week *in vivo* study of pMSC-seeded SIS-ECM in an epicardial location demonstrated that presence of MSCs significantly reduced T cell infiltration and associated adaptive immune response towards the biomaterial. Together, our *in vitro* characterization and *in vivo* application findings indicated that MSC-seeded SIS-ECM could serve as a promising biomaterial candidate for future cardiovascular applications.

Previous efforts using cell therapy have met with limited clinical success due to poor cell retention rate post-implantation or injection [43, 44]. A porous biomaterial [19], such as SIS-ECM, capable of supporting cell repopulation and growth has the potential to act as a delivery vehicle, improving cell retention rate *in vivo* while still allowing the repopulated cells to migrate within the local injury site. The maximum loading density of hMSCs on the FDA-approved porcine SIS-ECM material was found to be 350,000 cells per cm^2^ without affecting cell viability (Fig 1A). Establishing the maximum loading density on the matrix without inducing cell death is essential to establish maximal deliverable cell dosage in an hMSC-seeded SIS-ECM construct. More seeded MSCs could translate to more cells to be retained at the target site to increase its potential therapeutic effect. Once seeded, hMSCs were observed to maintain their morphology on SIS-ECM comparable to those grown in tissue-cultured wells (Fig 1B and 1D). These findings indicate the compatibility of SIS-ECM matrix with hMSCs, and promote its potential role as a patch vehicle allowing hMSC adherence during culture. Indeed, we observed that majority of seeded hMSCs adhered on the surface of SIS-ECM, while some migrated toward deeper layers of the porous SIS-ECM during short-term culture (Fig 2A). It remains to be determined if longer culture duration can increase the number of hMSCs migrating toward deeper layers of SIS-ECM. Examination of matrix topography with SEM imaging confirmed that collagen fibers and fibrils were intact and readily detectable throughout the decellularized porous SIS-ECM matrix (Fig 3A–3C). Seeded hMSCs were observed to attach and grow with cell extensions reaching out to surrounding collagen fibers and colonizing on the surface of SIS-ECM (Fig 3C and 3D). Together, our data supported that collagen-rich SIS-ECM was capable of hMSC recellularization *in vitro*, and seeded hMSCs were viable and adhered between collagen fibers. Such healthy cell-matrix interaction could facilitate *in vivo* translation of the repopulated SIS-ECM construct to achieve higher cell retention rate post-implantation in cardiovascular applications.

The mechanical properties of SIS-ECM are critical to its use as a clinical cardiovascular biomaterial. Previous literature reports indicate that porcine decellularized SIS-ECM is a biomaterial with high tensile strength [12, 45]. Indeed, the FDA-approved SIS-ECM has been applied as a sole graft material in various clinical surgeries repairing vascular, valvular and pericardial defects [12–18], indicating its capability to meet the functional requirements for cardiovascular tissues. Preservation of the mechanical integrity of SIS-ECM following cell seeding biomaterial is critical to guarantee the resultant construct will be able to endure the native cardiac tissue mechanical demands. Our findings confirmed that hMSC-seeding did not negatively affect the tensile properties of SIS-ECM (Fig 4), thus substantiating the potential of such seeded construct as a clinically relevant biomaterial for cardiovascular applications.

Preservation of biochemical composition of the matrix is critical to ensure the functional properties of the ECM for healthy cell-cell and cell-matrix interactions. Collagen and elastin are both major biochemical ECM components relevant in most tissues and organs. Our data showed that hMSC-seeding did not result in significant negative changes to collagen and elastin content of SIS-ECM (Fig 5A and 5C). Degree of collagen crosslinking was also preserved indicating that mature collagen fibers were not modified post-seeding (Fig 5B). Preservation of collagen content and crosslinking can be an important indicator of ECM health since breakdown or degradation of ECM collagen is typically associated with diseased or abnormal tissue functions [46]. Similarly, presence of elastin contributes to ECM elastic properties and its role is particularly crucial in cardiovascular tissues, such as blood vessels. There was a small amount of sGAG loss in SIS-ECM subjected to culture medium, regardless of hMSC-seeding (Fig 5D), suggesting that sGAG was capable of diffusing from SIS-ECM in culture. Since the amount of sGAG present (~2.5%) in decellularized SIS-ECM was initially quite small, the trivial sGAG loss observed in this study was unlikely to result in dramatic functional outcomes. As a soluble compound, sGAG is often found to be lost during culture in many engineering systems [47, 48]. Indeed, mechanical tensile testing showed that there was no functional difference across all groups (Fig 4), substantiating the preservation of mechanical properties even in the presence of small sGAG loss. Overall, we demonstrated that biochemical compositions of SIS-ECM were largely preserved following *in vitro* recellularization with hMSCs.

The translational potential and feasibility of the combined MSCs and SIS-ECM biomaterial for cardiovascular application were further validated by our two-week *in vivo* porcine study (Fig 7A). Our data showed that presence of MSCs significantly reduced graft-specific adaptive immune responses compared to SIS-ECM alone (Fig 7B). This finding was predominantly due to reduction in T cell response in all MSC-seeded groups, compared to SIS-ECM alone (Fig 7B). These findings suggest that the immunomodulatory properties of MSCs on SIS-ECM were retained and could be beneficial to mitigate *in vivo* adaptive immune responses. The effects of MSC dose (low vs. high) and seeding orientation (pericardial or epicardial) were not statistically significant based on both T cell and B cell quantifications (Fig 7B and 7C). Recent studies reported that hMSC cytokine secretion profiles (i.e vascular endothelial growth factor and interleukin-8) were increased when seeded on SIS-ECM compared to tissue culture plastic [37, 38]. These studies suggest a potential therapeutic benefit of hMSC-seeded SIS-ECM biomaterial via increased pro-angiogenic cytokine release. Qualitatively, CD31 staining was comparable across groups in the present study, suggesting that MSC-seeding failed to alter the ability of SIS-ECM biomaterial to integrate within the surrounding myocardium or to recruit new blood vessels. It is possible that the use of a non-injury model in the current work may have resulted in insufficient stimulus to promote MSC pro-regenerative function. Furthermore, due to the single time point utilized in the current study, we were unable to determine if SIS-ECM seeding resulted in enhanced retention of MSC at the implantation site over time. Consequently, future *in vivo* investigations in a chronic cardiac injury model (i.e myocardial infarct) are warranted to provide additional information regarding the efficacy of the combined biomaterial of SIS-ECM with MSCs in stimulating regenerative response.

## Conclusions

With the clinical success of decellularized porcine SIS-ECM as a graft material and the use of MSC as a promising cell therapy source for cardiac tissue repairs, harnessing the combined effects of an FDA-approved SIS-ECM product with MSCs may further enhance the clinical impact of the seeded biomaterial. This study demonstrates that FDA-approved porous decellularized porcine SIS-ECM can serve as a delivery vehicle for supporting MSC repopulation, and that such a seeded biomaterial has relatively intact structural, biochemical and mechanical properties *in vitro*. Lastly, seeded MSCs demonstrate similar properties pre- and post-seeding on SIS-ECM and may reduce *in vivo* graft-specific adaptive immune response for cardiovascular indications.

## Supporting Information

S1 Fig Dogbone Schematic for Mechanical Testing. A schematic illustration of dogbone shape with 2 mm gauge length used for mechanical testing. All SIS-ECM materials were prepared as strips (3 x 10 mm), and a dogbone neck region was created using a 2 mm biopsy punch.
(PDF)

S2 Fig Tri-lineage Differentiation of Porcine MSCs. Tri-lineage differentiations of pMSCs into adipocytes, chondrocytes and osteocytes were assessed before and after seeding on SIS-ECM.
(PDF)

S1 Table Patch Groups for Porcine *In vivo* Study. Groups of SIS-ECM patch alone, patches seeded with low vs. high dose of pMSCs and corresponding orientation for the porcine *in vivo* study.
(PDF)
