# Supplementary figures and images for: Mesenchymal Stem Cell Seeding of Porcine Small Intestinal Submucosal Extracellular Matrix for Cardiovascular Applications
Source: PLoS One. 2016 Apr 12;11(4):e0153412. doi: 10.1371/journal.pone.0153412 (PMC4829265; doi:10.1371/journal.pone.0153412)

**S1 Fig. Dogbone Schematic for Mechanical Testing.**

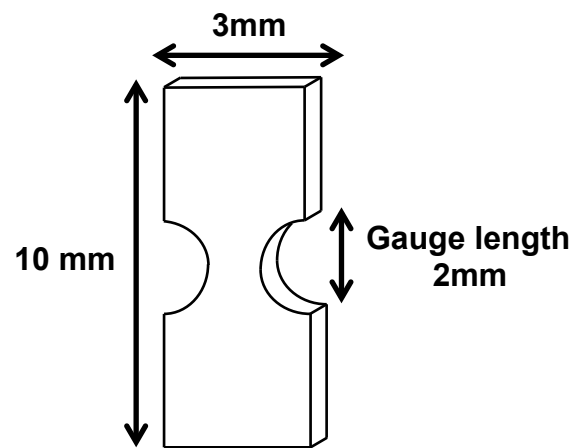

Supplement: S1 Fig — A schematic illustration of dogbone shape with 2 mm gauge length used for mechanical testing. All SIS-ECM materials were prepared as strips (3 x 10 mm), and a dogbone neck region was created using a 2 mm biopsy punch. (PDF) [file pone.0153412.s001.pdf]

**S2 Fig. Tri-lineage Differentiation of Porcine MSCs.**

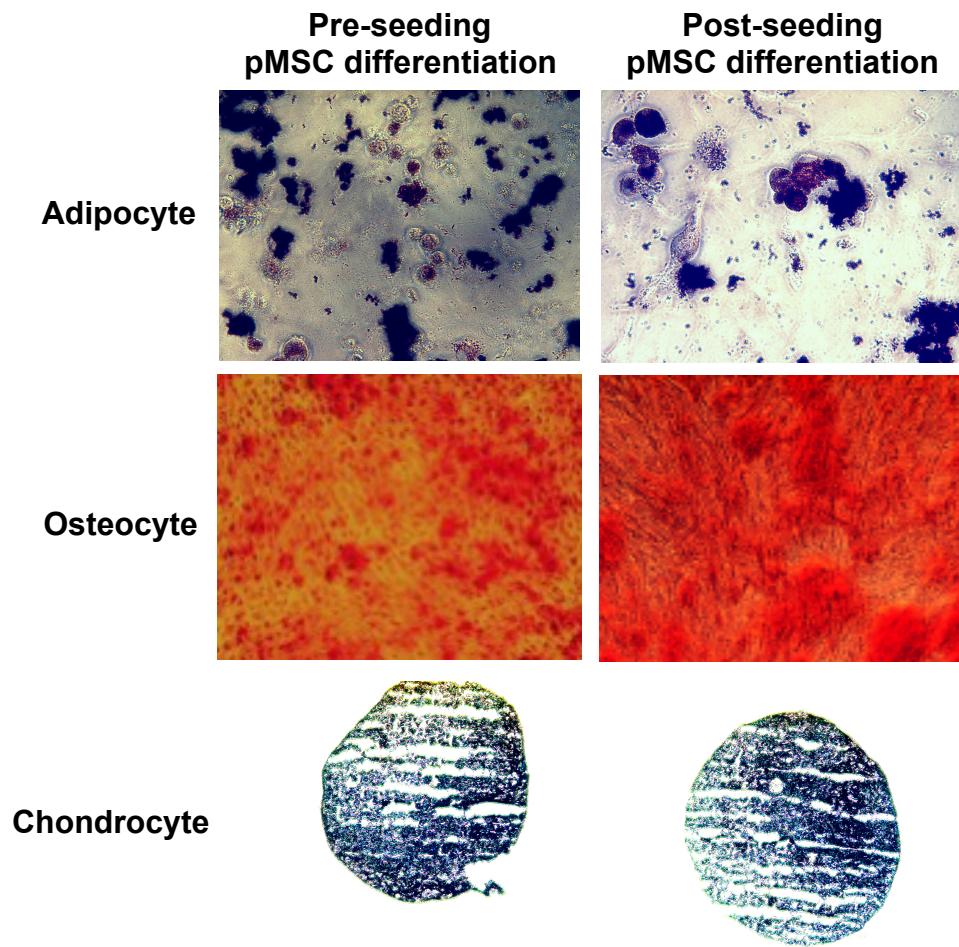

Supplement: S2 Fig — Tri-lineage differentiations of pMSCs into adipocytes, chondrocytes and osteocytes were assessed before and after seeding on SIS-ECM. (PDF) [file pone.0153412.s002.pdf]
