# Supplementary material for: Mesenchymal Stem Cell Seeding of Porcine Small Intestinal Submucosal Extracellular Matrix for Cardiovascular Applications
Source: PLoS One. 2016 Apr 12;11(4):e0153412. doi: 10.1371/journal.pone.0153412 (PMC4829265; doi:10.1371/journal.pone.0153412)
Supplement: S1 Table — Groups of SIS-ECM patch alone, patches seeded with low vs. high dose of pMSCs and corresponding orientation for the porcine in vivo study. (PDF) [file pone.0153412.s003.pdf]

S1 Table. Patch Groups for Porcine *In Vivo* Study.

Group	1	2	3	4	5
Porcine MSC dose (high vs. low)	No seeding	Low dose	Low dose	High dose	High dose
Patch orientation (pericardium vs. epicardium)	-	Facing pericardium	Facing epicardium	Facing pericardium	Facing epicardium
